# Retinopathy of prematurity and neurodevelopmental outcomes in preterm infants: A systematic review and meta-analysis

**DOI:** 10.3389/fped.2023.1055813

**Published:** 2023-03-15

**Authors:** Shivashankar Diggikar, Puvaneswari Gurumoorthy, Paula Trif, Diana Mudura, N. Karthik Nagesh, Radu Galis, Anand Vinekar, Boris W. Kramer

**Affiliations:** ^1^ Department of Paediatrics, Oyster Woman and Child Hospital, Bengaluru, India; ^2^ Centre for Cellular and Molecular Platforms, National Centre for Biological Sciences, Bengaluru, India; ^3^ Department of Neonatology, Emergency County Hospital of Bihor, Oradea, Romania; ^4^ Faculty of Medicine and Pharmacy, University of Oradea, Oradea, Romania; ^5^ Department of Neonatology, Manipal Hospitals, Bengaluru, India; ^6^ Department of Paediatric Retina, Narayana Nethralaya Eye Institute, Bengaluru, India; ^7^ Department of Paediatrics, School for Oncology and Developmental Biology, Maastricht University Medical Center, Maastricht, Netherlands; ^8^ School of Women’s and Infants’ Health, University of Western Australia, Crawley, WA, Australia

**Keywords:** retinopathy of prematurity, preterm, cerebral palsy, bevacizumab, ranibizumab, anti-VEGF, behavioural issues

## Abstract

**Background:**

Retinopathy of prematurity (ROP) and abnormal brain development share similar risk factors and mechanisms. There has been contrasting evidence on the association of ROP with adverse neurodevelopmental outcomes.

**Objective:**

We analysed the association between ROP at levels of severity and treatment with all neurodevelopmental outcomes until adolescence.

**Data source:**

We followed PRISMA guidelines and searched Medline and Embase between 1 August 1990 and 31 March 2022.

**Study selection and participants:**

Randomised or quasi-randomised clinical trials and observational studies on preterm infants (<37 weeks) with ROP [type 1 or severe ROP, type 2 or milder ROP, laser or anti-vascular endothelial growth factor (VEGF) treated] were included.

**Data extraction and synthesis:**

We included studies on ROP and any neurocognitive or neuropsychiatric outcomes.

**Outcomes:**

The primary outcomes were as follows: cognitive composite scores evaluated between the ages of 18 and 48 months by the Bayley Scales of Infant and Toddler Development (BSID) or equivalent; neurodevelopmental impairment (NDI; moderate to severe NDI or severe NDI), cerebral palsy, cognitive impairment; and neuropsychiatric or behavioural problems. The secondary outcomes were as follows: motor and language composite scores evaluated between the ages of 18 and 48 months by BSID or equivalent; motor/language impairment; and moderate/severe NDI as defined by the authors.

**Results:**

In preterm infants, “any ROP” was associated with an increased risk of cognitive impairment or intellectual disability [*n* = 83,506; odds ratio (OR): 2.56; 95% CI: 1.40–4.69; *p* = 0.002], cerebral palsy (*n* = 3,706; OR: 2.26; 95% CI: 1.72–2.96; *p* < 0.001), behavioural problems (*n* = 81,439; OR: 2.45; 95% CI: 1.03–5.83; *p* = 0.04), or NDI as defined by authors (*n* = 1,930; OR: 3.83; 95% CI: 1.61–9.12; *p* = 0.002). Type 1 or severe ROP increased the risk of cerebral palsy (OR: 2.19; 95% CI: 1.23–3.88; *p* = 0.07), cognitive impairment or intellectual disability (*n* = 5,167; OR: 3.56; 95% CI: 2.6–4.86; *p* < 0.001), and behavioural problems (*n* = 5,500; OR: 2.76; 95% CI: 2.11–3.60; *p* < 0.001) more than type 2 ROP at 18–24 months. Infants treated with anti-VEGF had higher odds of moderate cognitive impairment than the laser surgery group if adjusted data (gestational age, sex severe intraventricular haemorrhage, bronchopulmonary dysplasia, sepsis, surgical necrotising enterocolitis, and maternal education) were analysed [adjusted OR (aOR): 1.93; 95% CI: 1.23–3.03; *p* = 0.04], but not for cerebral palsy (aOR: 1.29; 95% CI: 0.65–2.56; *p* = 0.45). All outcomes were adjudged with a “very low” certainty of evidence.

**Conclusion and relevance:**

Infants with “any ROP” had higher risks of cognitive impairment or intellectual disability, cerebral palsy, and behavioural problems. Anti-VEGF treatment increased the risk of moderate cognitive impairment. These results support the association of ROP and anti-VEGF treatment with adverse neurodevelopmental outcomes.

**Systematic Review Registration:**

https://www.crd.york.ac.uk/prospero/, identifier: CRD42022326009.

## Introduction

Retinopathy of prematurity (ROP), a neurovascular disease caused by abnormal retinal vascularisation, is a complication after preterm birth that is still the most common cause of blindness in very preterm infants (1, 2). Retinal vascularisation begins around 12 weeks in utero and continues from the centre to the periphery until 44 weeks under the influence of vascular endothelial growth factor (VEGF). The pathogenesis of ROP involves the initial phase of vaso-obliteration of the retinal vasculature due to extrauterine hyperoxia, low levels of insulin-like growth factor 1 (IGF-1), and delayed expression of VEGF receptors. The next phase is characterised by vaso-proliferation secondary to the increased level of local VEGF levels (3–5). ROP is classified by four zones, five stages of severity, and the presence of plus disease, a posterior retinal vascular biomarker often warranting treatment (6, 7). As per the Early Treatment of Retinopathy of Prematurity Randomised (ETROP) trial, the disease is categorised into the following: type 1, defined as zone I, any stage with plus disease or zone I, stage 3 ROP without plus disease or zone II, or stage 2 or 3 ROP with plus disease; and type 2, defined as zone I, stage 1 or 2 without plus disease, or zone II stage 3 without plus disease (8). Several treatment approaches have been developed over time, aiming at the ablation of vessels by cryotherapy or laser photocoagulation to the avascular retina or intravitreal anti-VEGF injection within 48–72 h for type 1 ROP and close monitoring for type 2 ROP (8, 9).

Recent evidence has shown an association of severe ROP with adverse neurodevelopmental outcomes mainly in the cognitive component in preterm infants (10, 11). There are also scanty data on some correlation between ROP and behavioural problems, such as autism spectrum disorders (ASD) in extreme preterm infants attributed to poor brain growth (12, 13). The pathological process involved in ROP and abnormal neurodevelopmental outcomes share a common pathway (14). It could thus be plausible that ROP could be an independent biomarker for adverse neurodevelopmental outcomes (6). The long term follow-up concerning safety and efficacy is not fully understood, especially with the use of anti-VEGF. The epoch of development and treatment of ROP coincides in principle during late pregnancy, when exponential growth of the brain occurs. This growth is only possible with appropriate growth of the microvasculature. We have little information about how development of the aberrant retinal microvasculature in ROP also affects other parts of the brain during this particular phase of exponential brain growth (15–17). This raises the question about the association between ROP, treatment of ROP, and the infant’s neurodevelopmental outcome (18, 19).

We performed a systematic review and a meta-analysis to ask the following three questions: Is there a correlation between ROP and short- or long-term neurodevelopmental outcomes? If so, can we identify a threshold of the severity of ROP disease that is associated with subsequent impaired neurodevelopmental outcome? And third, is there a difference in the association depending on the type of treatment?

## Methods

The present systematic review was conducted according to Preferred Reporting Items for Systematic Reviews and Meta-analyses (PRISMA) reporting guidelines (20). In addition, we developed the protocol *a priori*, which specified the inclusion criteria, the method for evaluating study quality, outcomes, and statistics. The protocol was registered with the international prospective register for systematic reviews, PROSPERO (CRD42022326009).

### Search strategy

A systematic literature search was conducted using an appropriate prespecified search strategy in Ovid Medline and Embase between 1 August 1990 and 31 March 2022, using Medical Subject Headings. Details of the search strategy are provided in the Supplementary Material.

### Study selection

Randomised or quasi-randomised clinical trials and observational studies that evaluated at least one of the prespecified outcomes were included. Preterm infants (<37 weeks) with any ROP (type 1 or severe ROP, type 2 or milder ROP, laser or anti-VEGF treatment) were included. Preterm infants with genetic syndromes and congenital anomalies were excluded. Preterm infants without ROP were considered to be comparators.

### Outcomes

#### Primary outcomes

(1) Cognitive Composite Scores (CCS) evaluated between 18 and 24 months of age by the Bayley Scale of Infant and Toddler Development (BSID III/IV) or equivalent; between 25 and 48 months if reported.
(2) Neurodevelopmental impairment (NDI) as defined:
  (a) Moderate to severe NDI, defined as the presence of one or more of the following: BSID III/IV (cognitive, motor, or language score) <85, cerebral palsy (CP), visual impairment (unilateral or bilateral blindness), or severe to profound hearing impairment (meeting criteria for amplification) evaluated between 18 and 48 months of age.
  (b) Severe NDI, defined as the presence of one or more: BSID III/IV (cognitive, motor, or language score) <70, CP with a Gross Motor Functional Classification Scale (GMFCS) level ≥3, blindness (bilateral blindness with or without some functional vision in one or both eyes), or severe to profound hearing impairment (requiring cochlear implants in one/both ears or permanent hearing loss that prevented the understanding of instructions) evaluated at 18–48 months of age.
(3) CP (any type) evaluated clinically between 18 and 48 months of age.
(4) Cognitive impairment (6 months to 21 years): moderate (BSID-III < 85) or severe (BSID-III < 70) or defined by any comparable validated tool.
(5) Neuropsychiatric or behavioural problems (attention deficit hyperactivity disorder, ASDs, or others) evaluated by any validated tool.

#### Secondary outcome(s)

(1) Motor and language composite scores evaluated between 18 and 48 months of age by BSID-III/IV or any validated tool.
(2) Motor impairment evaluated between 18 and 48 months of age: moderate (BSID-III < 85) or severe (BSID-III < 70) or defined by any comparable validated tool.
(3) Language impairment evaluated between 18 and 48 months of age: moderate (BSID-III < 85) or severe (BSID-III < 70) or defined by any comparable validated tool.
(4) Motor function evaluated above 4 years of age using any validated tool.
(5) Moderate or severe NDI as defined by the authors.

### Data extraction (selection and coding)

Two authors (SD and PG) searched the databases per a predefined search strategy. The final articles were compiled and transferred to Rayyan software (www.rayyan.ai) and the duplicates were removed. Title and abstract screening and full-text screening of articles were done independently by SD/BK. Any discrepancy was resolved by discussion with all the authors. All authors agreed with the final list of articles. The trial authors were contacted by email correspondence to request missing data if needed. The discrepancies were resolved by discussion and consensus with authors BK/NN/AV.

### Assessment of methodological quality

All included studies were assessed for methodological quality. The risk of bias was assessed using elements of the Cochrane Collaboration tool for randomised studies (21). For observational studies, the risk of bias for included studies was assessed using a modified Newcastle-Ottawa Scale (NOS) (22) and the following domains were evaluated: selection; comparability; and outcome. A priori, a score of >7/9 was deemed low risk, a score of 4–6/9 was deemed a moderate risk, and a score of ≤3/9 was deemed a high risk of bias. Two authors (SD and PG) performed the risk of bias independently; conflicts were resolved after discussion and consensus with other authors (BK and NN). Similarly, two authors (SD and PG) assessed the certainty of evidence (confidence in the estimate of effect) for each outcome based on the Grading of Recommendations Assessment, Development, and Evaluation (GRADE) framework (23). Any discrepancy arising out of subjective assessments was resolved by discussion and consensus.

### Data synthesis and statistical analysis

All the studies were combined and analysed using Comprehensive Meta-Analysis version 3.0 software (Biostat Inc., Boston, MA, USA). For continuous outcomes, the mean difference with 95% CI was calculated and for dichotomous outcomes, the odds ratio (OR) with 95% CI was calculated from the data provided in the studies. Adjusted ORs (aORs) for potential confounders were extracted from the studies reporting these data. Studies reporting continuous variables as median and range or interquartile range were converted to mean and SD using the published calculator (24). A random effects model was used to calculate the summary statistics owing to anticipated heterogeneity. For some variables, such as gestational age (GA), a fixed outcome model was used. Statistical heterogeneity was assessed by using the Cochran *Q* statistic and the *I*^2^ statistic, which is derived from the *Q* statistic and describes the proportion of total variation that is due to heterogeneity beyond chance. We used the Egger regression test and funnel plots to assess publication bias. GA as a potential source of variability between the groups was identified *a priori* and was included for meta-regression (25).

## Results

**Figure 1:**
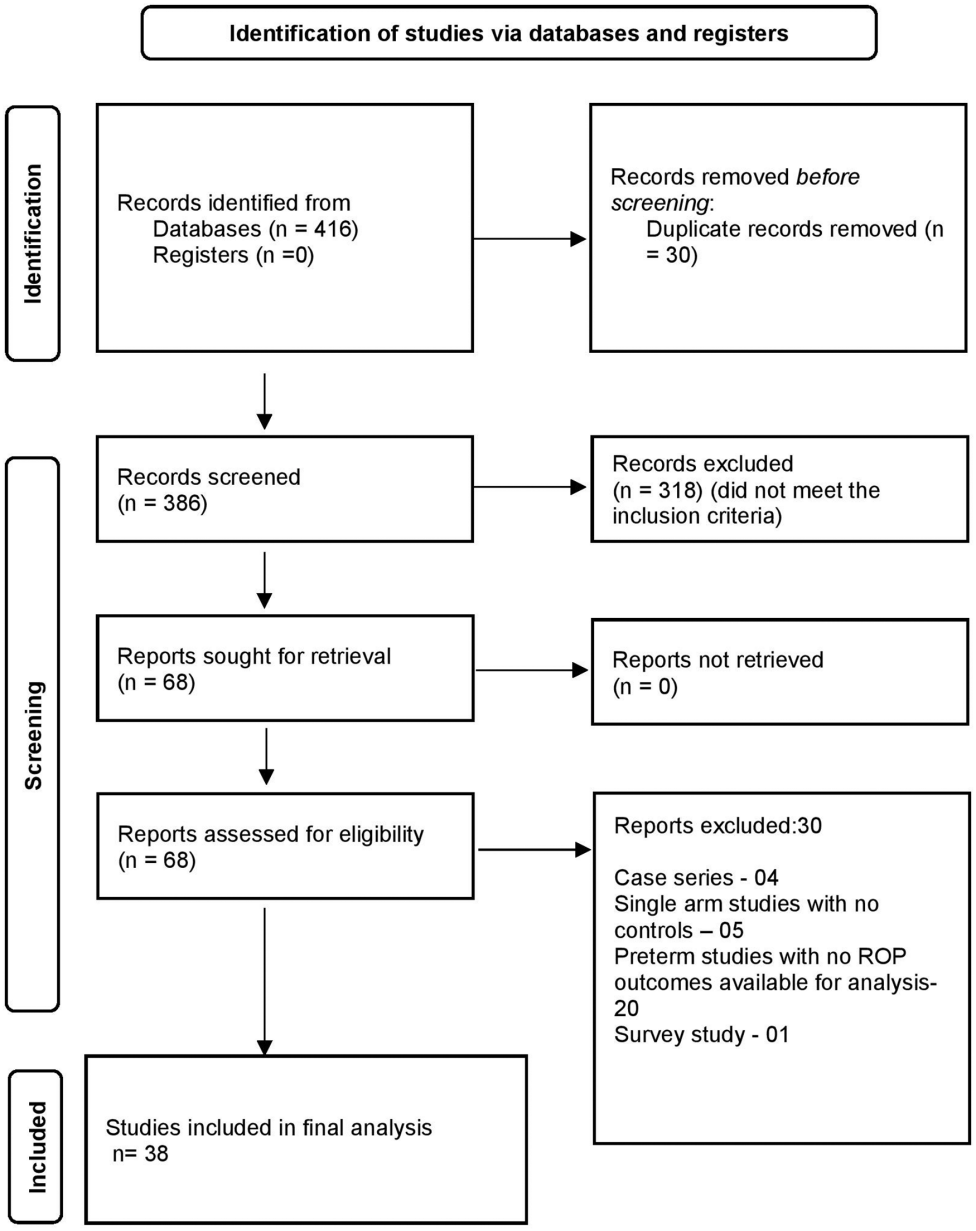
Flowchart of search results (adapted from PRISMA 2021).

A total of 416 articles were identified through all databases, of which 386 articles underwent title and abstract screening. There were 68 full-text articles assessed for eligibility. Finally, 38 articles were deemed eligible for inclusion in the analysis. The selection process of articles and final inclusion as per PRISMA guidelines (20) is provided in Figure 1.

### (a) Any ROP vs No ROP

A total of 22 studies (10, 11, 26–45) reported on neurodevelopmental outcomes (includes all outcomes) between “no ROP” and “any ROP.”

**Figure 2:**
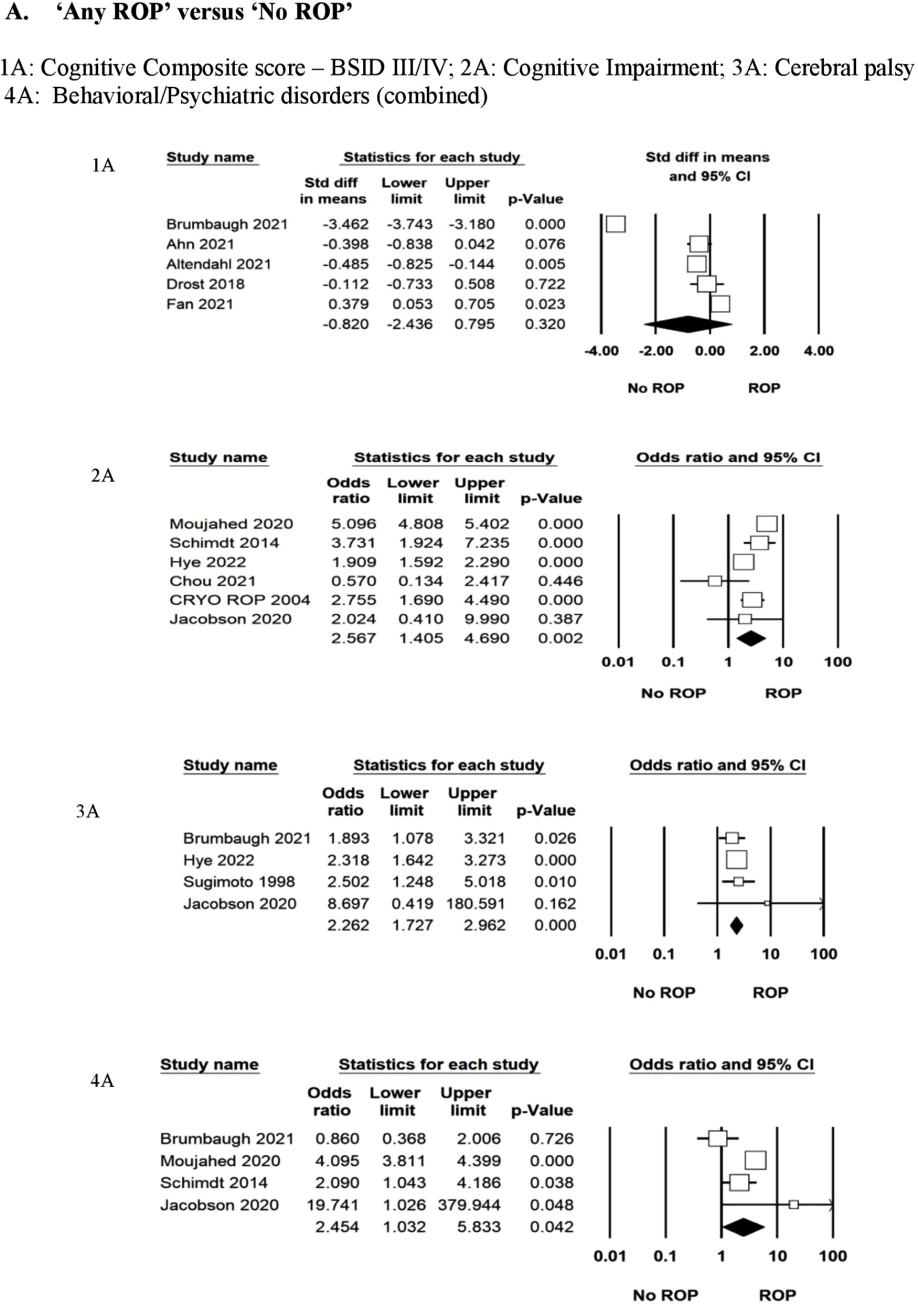

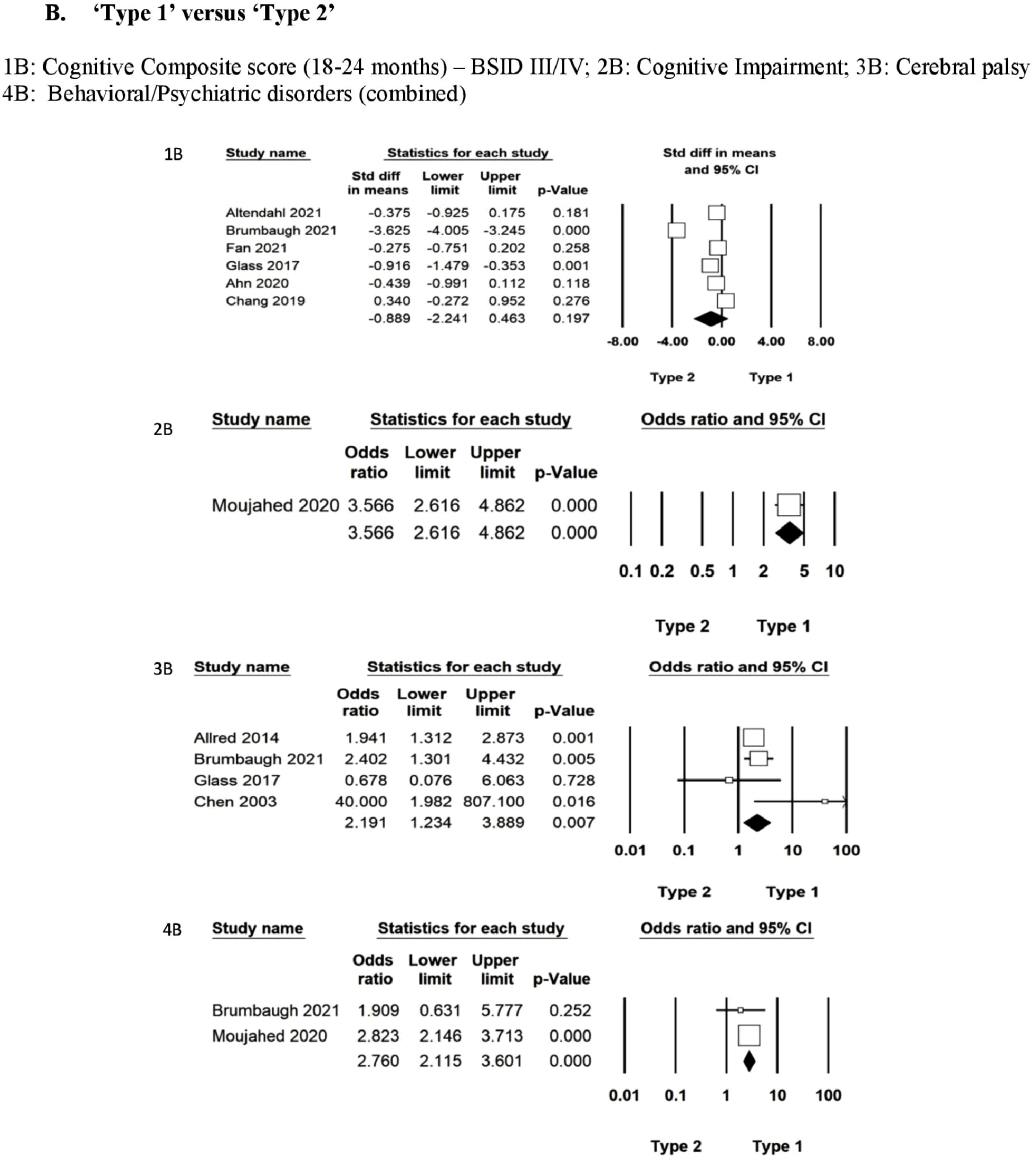

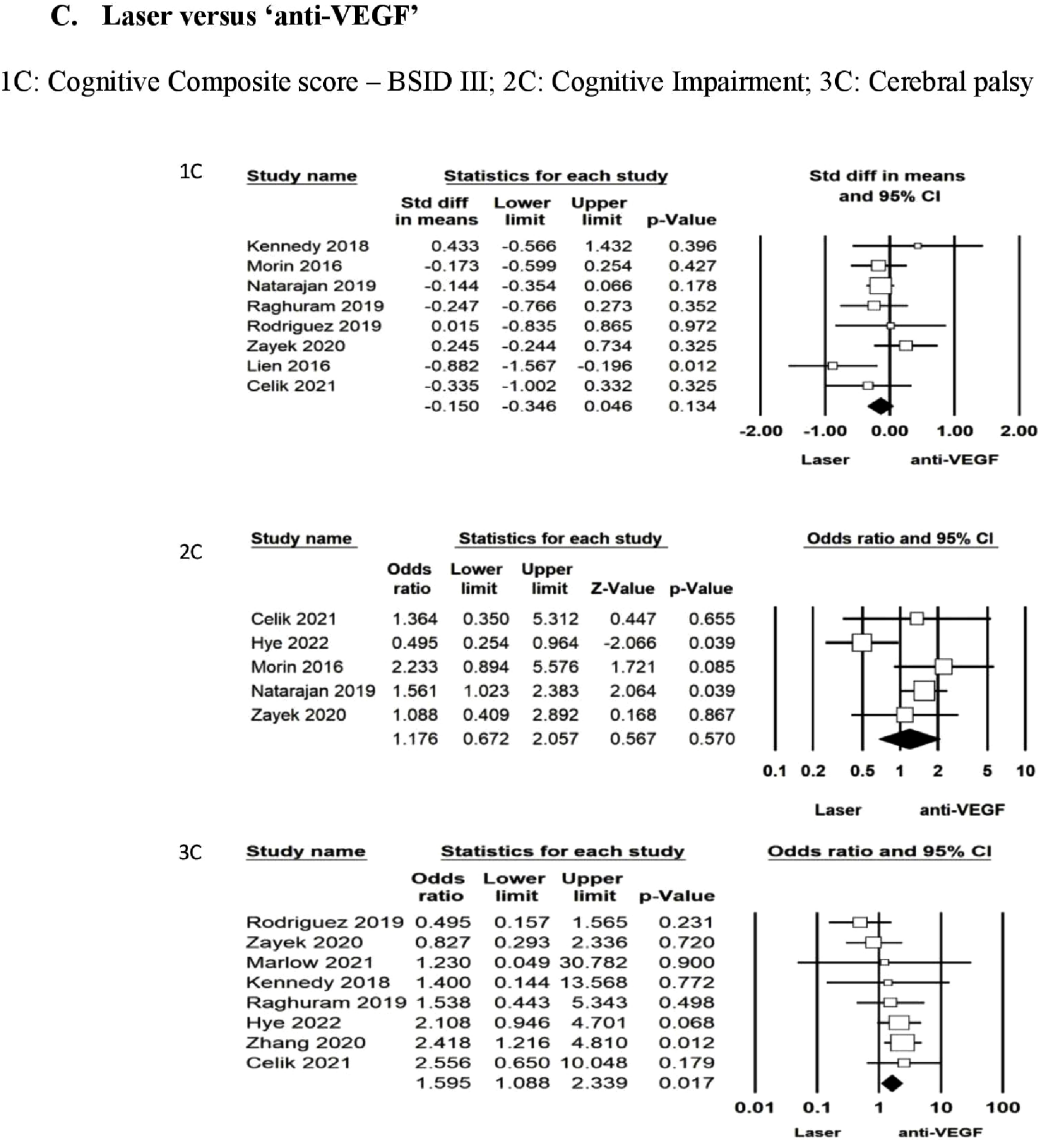
Forest plots for primary and important secondary long term neurodevelopmental outcomes.

The primary outcome of CCS on BSID-III/IV was reported in five studies (*n* = 922) at 18–24 months (26, 28, 34, 35, 44). The standard mean difference (SMD) was not different between the “no ROP” and “any ROP” groups (SMD: −0.820 to −2.43; *p* = 0.32; *I*^2^=98%). Data were inadequate to pool for the meta-analysis for moderate or severe NDI. Cognitive impairment/intellectual disability as defined by the author using different scales was reported in six studies (10, 30, 33, 37, 41, 43). A total of 83,506 infants were included in the analysis, which showed significantly increased odds in the “any ROP” group (OR: 2.56; 95% CI: 1.40–4.69; *p* = 0.002; *I*^2^=95%). CP was reported in four studies (38, 41, 43, 44) (*n* = 3,706), which showed increased odds of CP (OR: 2.26; 95% CI: 1.72–2.96; *p* < 0.001; *I*^2^=0%) in the “any ROP” group. Behavioural or neuropsychiatric problems as defined by the authors were reported in four studies (*n* = 81,439) (10, 37, 41, 44). Two studies used the Child Behaviour Check List (10, 44) and one study (37) used the International Classification of Disease codes (ICD), whereas another study (41) used the Swedish questionnaire to define the problem between the ages of 2 and 18 years. There was a statistically significant difference with increased odds in the “any ROP” group (OR: 2.45; 95% CI: 1.03–5.83; *p* = 0.04; *I*^2^=83%) (Figure 2 (1A, 2A, 3A, 4A)).

Secondary outcomes of language and motor composite score (BSID-III/IV) were reported in four studies (*n* = 877) (26, 28, 35, 44) at 18–24 months. The SMD favoured the “No ROP” group for both domains (SMD: −0.73 to −0.15; *p* = 0.002; *I*^2^=70% and SMD: −0.46 to −0.11; *p* = 0.001; *I*^2^=28%, respectively). NDI as defined by authors was reported in five studies (*n* = 1,930) (29, 31, 36, 42, 45). The age at which NDI was defined varied from 3 months to 7 years of life. “Any ROP” increased the odds of NDI significantly (OR: 3.83; 95% CI: 1.61–9.12; *p* = 0.002; *I*^2^=72%) (Supplementary Figures).

### (b) Type 1 vs Type 2

A total of 11 studies (26–28, 34, 35, 44, 46–50) reported data between mild and severe forms of ROP. Six studies (28, 35, 43, 44, 47, 49) (*n* = 689) reported on the primary outcome of CCS measured by BSID-III/IV between the ages of 18 and 24 months. The results were not statistically significant between the groups (SMD: 0.88 to −2.24; *p* = 0.19; *I*^2^=97%). Four studies (*n* = 1,517) reported CP (44, 46–48). Type 1 or severe ROP increased the risk of CP (OR: 2.19; 95% CI: 1.23–3.88; *p* = 0.07; *I*^2^=40%) twofold compared to type 2 ROP at 18–24 months. Cognitive impairment or intellectual disability was reported in one study (*n* = 5,167) (37). The odds for cognitive impairment were increased in type 1 ROP (OR: 3.56; 95% CI: 2.6–4.86; *p* < 0.001). Behavioural or neuropsychiatric problems were favouring type 2 ROP significantly (two studies, *n* = 5,500; OR: 2.76; 95% CI: 2.11–3.60; *p* < 0.001; *I*^2^=0%) (37, 44). Moujahed et al. (37) compared treated vs. not treated, which for study purposes we used as type 1 and type 2 for analysis. Cognitive impairment (BSID III < 85) or studies of moderate to severe NDI or severe NDI were not enough to pool for the analysis (Figure 2 (1B, 2B, 3B, 4B)).

Secondary outcomes from six studies (*n* = 687) of motor (SMD: −2.46 to 0.49; *p* = 0.19; *I*^2^=98%) and language composite score (SMD: −1.90 to 0.60; *p* = 0.31; *I*^2^=98%) were not different between the two groups (26, 28, 35, 44, 47, 49). For other outcomes, the number of studies was insufficient to pool for meta-analysis (Supplementary Figures).

### (c) Anti VEGF vs Laser

A total of 15 studies (30, 43, 51–63) reported the outcomes for anti-VEGF vs. laser (Bevacizumab 14 studies, Ranibizumab 1 study) and were included in the analysis. The primary outcome of CCS measured by BSID-III/IV or any other validated tool between 18 and 24 months was reported by nine studies (*n* = 803) (51–54, 56, 58, 60, 62, 63). One study used a different scale for assessment (51). The analysis was performed separately for different scales using the BSID-II/III, Kyoto Scale of Psychological Development (KSPD), and combined (Supplementary Material). There was no heterogeneity between studies (eight studies, *I*^2^=0%). The pooled size of the effect estimate was not significant (SMD: −0.34 to 0.04; *p* = 0.13; *I*^2^=0%). CP was reported in eight studies (*n* = 965) (43, 52–57, 60). There was statistical significance noted with the anti-VEGF group having higher odds of CP than laser surgery both in the random effects (OR: 1.55; 95% CI: 1.02–2.36; *p *= 0.04; *I*^2^=11%) and the fixed effect models (OR: 1.59; 95% CI: 1.08–2.33; *p* = 0.01). Cognitive impairment (BSID III/IV score <85 or any validated scale) was not different between the two groups (five studies, *n* = 834, OR: 1.17; 95% CI: 0.67–2.05; *I*^2^=60%) (43, 52, 53, 58, 63) (Figure 2 (1C, 2C, 3C)).

The secondary outcome of language composite score evaluated by BSID III/IV or any other validated scale was not different between the two groups (SMD: −0.22 to 0.08; *p* = 0.35; *I*^2^=0%) from eight studies (*n* = 748) (one study used KSPD, analysed separately) (52–54, 56, 58, 60, 62, 63). Moderate (BSID-III/IV < 85) (two studies, *p* = 0.66; *I*^2^=39%) or severe language impairment (BSID-III/IV < 70) (two studies, *p* = 0.77; *I*^2^=39%), as defined, was not different between the two groups. The motor composite score was not significantly different (nine studies, *n* = 792, SMD: −0.43 to 0.03; *p* = 0.08; *I*_2_=40) between the two groups from eight studies that used the BSID for assessment were analysed separately and there was no difference in outcome either (Supplementary Material). Moderate (BSID-III/IV < 85) (four studies, OR: 1.26; 95% CI: 0.91–1.75; *p* = 0.14; *I*^2^=0%) or severe motor impairment (BSID-III < 70) (two studies, OR: 1.10; 95% CI: 0.71–1.68; *p* = 0.66; *I*^2^=0%) were not different between the two groups. Five studies reported on moderate or severe NDI (*n* = 316, OR: 1.34; 95% CI: 0.77–2.32; *I*^2^=0%) and severe NDI (*n* = 681, OR: 1.39; 95% CI: 0.85–2.2; *I*^2^=39%), as defined; the results were not different between the groups (52, 53, 58, 60, 63). We tested whether the combined effect of “anti-VEGF plus laser” was less favourable for neurodevelopmental outcomes compared to “anti-VEGF.” Studies were not adequate for any conceivable conclusion or analysis (30, 52, 62). We analysed adverse outcomes (any) vs. no adverse outcomes due to the paucity of data for various outcomes to be combined. There was no difference observed between the two groups (Supplementary Material). A summary of all included studies is provided in Table 1.

**Table 1:** Summary of all included studies.

Study ID	Study type	Sample size (*n*=)	Domains assessed for neurodevelopment	Developmental scale	Age of assessment	Severe ROP definition	NOS Score
**Any ROP vs. No ROP**							
Joon Ahn 2021	Prospective	*n* = 81 (39/42)	Cognitive, language, motor composite scores	BSID-III	18 months	Severe ROP was defined as a stage ≥ 3 ROP	6
Altendahl 2021	Retrospective	*n* = 228 (117/74)	Cognitive, language, motor composite scores	BSID-III	0–12, 12–24, 25–36 months	As per ETROP study	7
Bohm 2002	Prospective	*n* = 145* (85/60)	Full scale, performance scale and verbal scale (IQ)	WPPI-R	5–6 years	ROP 1-stage 1–3 ROP 2-stage 3+	5
Beligere 2015	Prospective	*n* = 74 (61/13)	Multiple domains	Oregon Project skills inventory	3 months-5.5 years	As per ICROP	7
Chou 2021	Prospective	*n* = 207 (186/101)	Cognitive, language, motor composite scores,	BSID-II/III WPPSI-IV	4–6 years	As per ETROP study	8
CRYO-ROP 2001	RCT	*n *= 244 (142/102)	Cognition, emotion, hearing, speech	Health Utilities Index (HUI)—HRLQ score	10 years	NI	NA
Drost 2018	Retrospective	*n *= 36 (18/2)	Locomotor function, personal social functioning, hearing and language, eye and hand coordination, and performance	Griffiths Mental Development Scales BSID-III	15–24 months	ROP treated with laser therapy, and included grade 3 with plus disease or type 1 ROP	6
Fan 2019	Prospective	*n *= 148 (69/79)	Cognitive, language, motor composite scores Severe NDI	BSID-III	1–3 years	As per ETROP study	7
Hungerford 1986	Prospective	*n *= 177 (14/163)	Cerebral palsy and overall neurodevelopmental delay	NI	12–18 months	NI	5
Moujahed 2020	Retrospective Data base study	*n *= 79,373 (5,167/74,206)	Intellectual disabilities, speech and language, motor deficits, psychiatrics and behavioural problems	ICD code were used	Within 12 and 24 months	NI	7
Ricci 2020	Prospective	*n *= 105 (63/42)	Developmental quotient	Griffith's Mental Development Scales	24 months	As per ETROP study	6
Stephenson 2007	Prospective	*n *= 198 (106/92)	General conceptual ability, verbal and non-verbal reasoning cluster, spatial ability cluster, diagnostic scales	British Ability Scales II (BAS),	11–14 years	Stage 3 or worse	7
Sugimoto 1997	Retrospective	*n *= 1,081 (100/491)	Cerebral palsy and mental retardation	Clinical	10, 18 and 36 months	As per ICROP classification	5
Bae 2021	Retrospective	*n *= 240 (195/45)	Cerebral palsy (CP), hearing impairment and blindness	BSID-III	18–24 months	Those with a treatment threshold in stage III or higher, or in rapidly progressive disease requiring laser photocoagulation	7
Borregas 2018	Retrospective	*n *= 1,001 (40/961)	Motor impairment, severe cognitive impairment, GMFCS	NI	7 years	Stage 4 or 5	5
Chou 2021	Prospective	*n *= 64 (9/55)	Full-scale IQ	WPPI	3–6 years	As per ETROP study	8
Holsti 2018	Database study	*n *= 140/142 (132/134)	Neurosensory and cognitive delay	WISC-III	10–15 years	Bilateral disease of more than stage 3 or receiving retinal therapy	6
Hye 2022	Prospective	*n*= 2,132 (778/1,354)	Motor/cognitive/speech /CP/hemiplegia	BSID-II/III or K-DST	18–24 months	As per ICROP 2003/05	8
Todd 2012	Prospective	*n *= 272 (68/204)	Locomotor, personal–social, hearing and speech eye–hand coordination, performance, practical reasoning, general quotient	Griffiths Mental developmental scale	3 years	NI Stage 4/5 were excluded	6
Schmidt 2014	RCT	*n *= 1,582 (95/1,482)	Cognitive, motor and behavioural problems	WPPI-III, CBCL, GMFCS	5 year	Unilateral or bilateral stage 4/5 or needing treatment in at least one eye	NA
Jacobson 2020	Prospective	*n *= 39 (19/20)	Cognitive, mental health, CP	WISC-III	18 years	ROP stage 3 or more	7
Msall 2000	Prospective	*n *= 1,063 (713/350)	Functional independence measure (FIM) Severe disability	WeeFIM	5.5 years	NI	7
**Type 1 (severe forms) vs. Type 2 (milder forms)**							
Joon Ahn 2020	Prospective	*n *= 81 (16/65)	Cognitive, language, motor composite scores	BSID-III	18 months	Sage ≥ 3 ROP	6
Allred 2014	Prospective	*n *= 1,085 (305/780)	MDI and PDI, CP	BSID-II	24 months	As per ETROP study	7
Altendahl 2021	Retrospective	*n*= 74 (19/55)	Cognitive, language, motor composite scores	BSID-III	0–12, 12–24, 25–36 months	As per ETROP study	7
Bohm 2002	Prospective	*n* = 60* (20/40)	Full scale, Performance scale and Verbal scale (IQ)	WPPI-R	5–6 years	ROP 1-stage 1–3) ROP-stage 3+	5
Brumbaugh 2021	RCT	*n *= 333 (71/262)	Cognitive, Language, Motor composite scores CP, GMFCS/=2, ASD	BSID-III, CBCL	22–26 months	Severe ROP was defined as ROP needing intervention	NA
Glass 2017	Prospective	*n *= 83 (16/67)	Cognitive, Language, Motor composite scores, CP	BSID-III	18 months	As per ETROP study	7
Jin Choi 2022	Retrospective Database study	*n *= 6,995 (276/6,719)	Neurocognitive, speech and language, motor function developmental disorder (ICD codes)	ICD code were used	Upto 10 years	Severe ROP was defined as ROP needing treatment (as per ETROP study)	6
Moujahed 2020	Retrospective Database study	*n *= 5,167 (222/4,945)	Intellectual disabilities, speech and language, motor deficits, psychiatric and behavioural problems	ICD code were used	Within 12 and 24 months	NI	7
Chang 2019	Retrospective	*n *= 104 (18/86)	Cognitive, language, motor composite scores, NDI	BSID-II/III	6 month, 1 and 2 year	As per ETROP study	7
Chen 2003	Retrospective	*n *= 16 (5/11)	CP	NI	17 months	As per ETROP study	7
**Laser vs. anti-VEGF**							
Arima 2020	Retrospective	*n *= 53 (L/B) (39/14)	Postural-movement, cognitive-adaptive, or language-social domain	KSPD	18 months	As per ICROP 2003/05	6
Celik 2021	Retrospective	*n *= 54 32/12/10 (L/B/C)	Cognitive, language, motor NDI/sNDI, CP	BSID-III	12–42 months CA	As per ICROP 2003/05	8
Da Chou 2021	Prospective	*n *= 37 (5/23/9)	Full scale IQ	WPPI	3–6 years	As per ETROP study	8
Hye Ahn 2022	Retrospective	*n *= 778 (subgroup data available)	Motor/cognitive/speech /CP/hemiplegia	BSID-II/III or K-DST	18–24 months	As per ICROP 2003/05	8
Kennedy 2017	RCT	*n* = 16 (9/7)	Cognitive, language, motor CP, GMFCS	BSID-III	18–24 months	Stage 3+ ROP in zone I or zone II posterior in both eyes	NA
Lien 2016	Retrospective	*n*= 61 33/12/16	MDI and PDI	BSID-II	6, 12, 18, and 24 months	As per ETROP study	7
Marlow 2021	RCT	44/106 (Ranibizumab)	Receptive and expressive language, CP, GMFCS,	Mullen’s scale of early learning	20–28 months	Bilateral ROP zone I stage 1+, 2+, 3, or 3+, or zone II stage 3+, or aggressive posterior ROP.	NA
Morin 2016	Retrospective	*n *= 125 (98/27)	Cognitive, language, motor scores NDI/sNDI, CP	BSID-III	18 months	As per ETROP study	6
Murakami 2021	Retrospective	*n *= 26 (14/12)	DQ/IQ	WISC/KSPD	5 years	As per ETROP study	6
Natarajan 2019	Retrospective	*n *= 405 (224/181)	Cognitive, language, motor NDI/sNDI	BSID-III	18–26 months	Pragmatic definition of severe ROP	7
Raghuram 2019	Retrospective	*n *= 64 (30/34)	Cognitive, language, motor NDI/sNDI	BSID-III	18–24 months	As per ETROP study	7
Rodriguez 2019	Retrospective	*n *= 86 (40/46)	CP	BSID-III/GMA	Not defined	As per ETROP study	7
Zayek 2020	Retrospective	*n *= 116 (85/31)	Cognitive, language, motor NDI/sNDI, CP	BSID-III	18–24 months	As per ETROP study	7
Zhang 2020	Retrospective	*n *= 298 (235/63)	CP, cognitive/speech/motor delay	Database-ICD codes	NA	NI	7
Chen 2017	Retrospective	*n *= 25 (15/10)	neurodevelopmental delay (not defined)	Database-ICD codes	20 months	NI	7
**Others [anti-VEGF vs. (laser + anti-VEGF) OR laser vs. (laser + anti-VEGF)]**							
Araz-Ersan 2014	Prospective	*n *= 13	Cognitive, language, motor scores	BSID-III	24 months	As per ETROP study	5
Ahmed 2020	Retrospective	*n *= 66 (18-combined 48-Laser)	Cognitive, language, motor scores Developmental delay	BSID-III	24 months	As per ETROP study	5

L, laser; B, bevacizumab; C, combined (laser + bevacizumab); BSID, Bayley scale of infant and Toddler development; KSPD, Kyoto Scale of Psychological Development; GMFCS, gross motor functional classification system; CP, cerebral palsy; MDI, mental developmental index; PDI, psychomotor developmental index; K-DST, Korean development screening test; GMA, general movement assessment; WPPI-R, Weschler preschool and primary scale of intelligence—revised; CBCL, child behaviour checklist; HRQL, health-related quality of life; WISC, Weschler intelligence scale for children-III; sNDI, severe neurodevelopmental impairment; ETROP, early treatment for retinopathy of prematurity; ICROP, international classification for retinopathy of prematurity; ICD, international classification of diseases; IQ, intelligent quotient; NOS, New Castle-Ottawa scale; VEGF, vascular endothelial growth factor; WeeFIM, functional independence measure for children.

* (*n*=) is different for different domains, so an average of all scale is presented in the table.

### Meta-regression and adjusted analysis

The outcomes of CP and cognitive, language, and motor composite scores were adjusted by meta-regression with the GA as the confounding factor. GA did not account for the differences noted between the groups (Supplementary Material).

**Figure 3:**
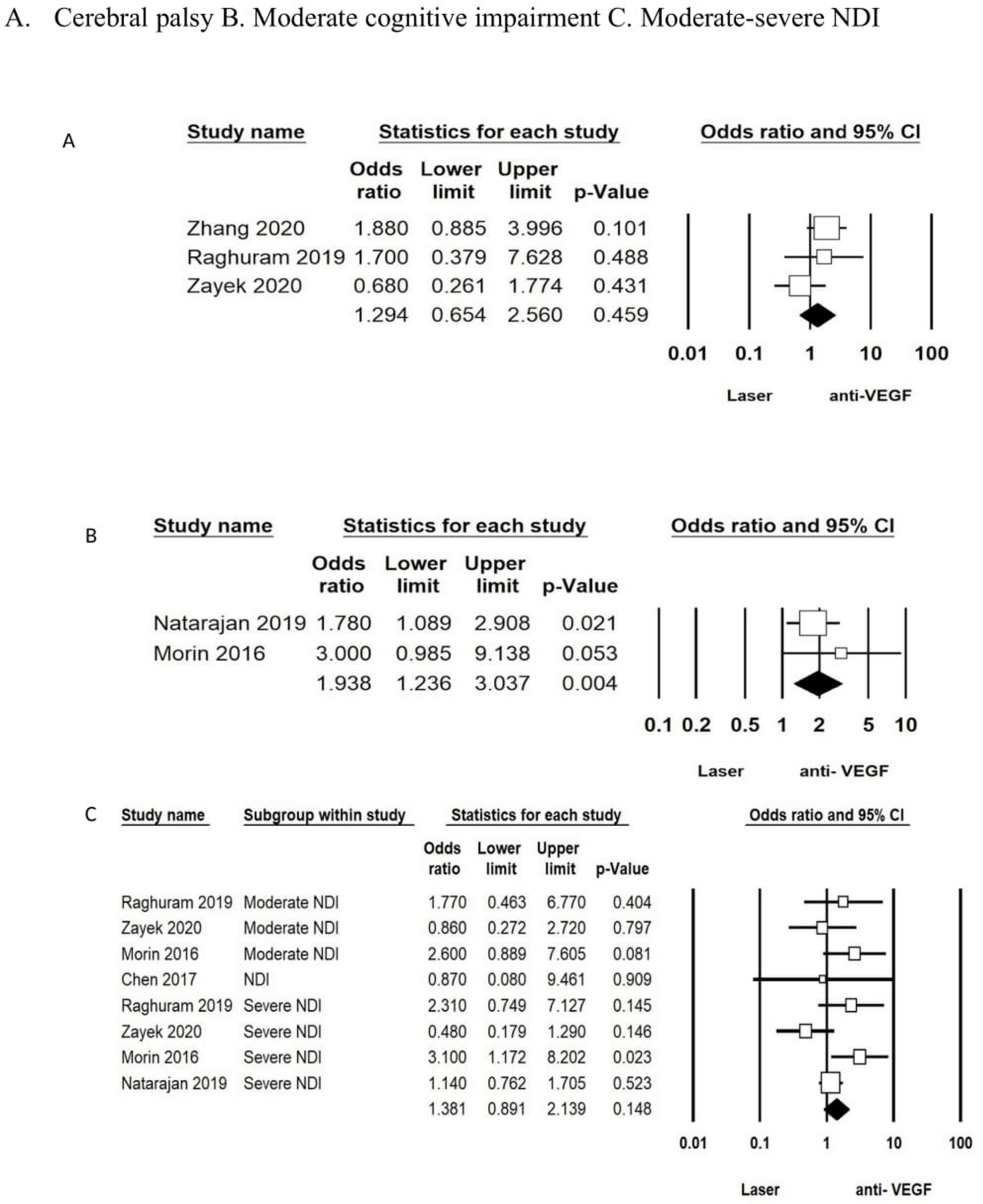
Forest plots: for adjusted OR analysis—laser vs. “anti-VEGF”.

The analysis for the studies that adjusted for comorbidities (IVH, white matter injury, surgical NEC, BPD), GA, and sex was conducted using the aORs. Two studies (58, 63) reported on aOR in the laser vs. anti-VEGF group and showed an increased risk for moderate cognitive impairment in the anti-VEGF group (aOR: 1.93; 95% CI: 1.23–3.03; *p* = 0.04). There was no difference for CP after adjusting for confounding variables (aOR: 1.29; 95% CI: 0.65–2.56; *p* = 0.45) between the two groups reported in three studies (53, 55, 60). The combined outcome of moderate or severe NDI or NDI as defined by the authors from five studies (53, 58, 60, 61, 63) was also not different between the groups (aOR: 1.38; 95% CI: 0.89–2.13; *p* = 0.14) (Figure 3 (A, B, C)).

### Risk of bias assessment and certainty of evidence

The risk of bias assessment was performed as per the ROB.2 tool (21) for randomised controlled trials and NOS for observational studies (22). Most of the studies were of fair or good quality. Three randomised controlled trials (10, 44, 57) were considered to be with a “low risk” of bias and two studies (33, 54) with a “high risk” of bias (Supplementary Material). The certainty of evidence was graded as “very low” for all the outcomes (Table 2).

**Table 2:** GRADE summary of findings (SoF).

Sino	Outcomes	No. of studies	Predominant studies included	No. of neonates evaluated	Anticipated absolute effects—(95% CI)		Relative effect (95% CI)	Certainty of the evidence (GRADE)
**“No ROP” vs. “any ROP”**								
	Risk* with “No ROP”	Risk with “Any ROP”		
1	Cognitive Impairment or Intellectual Disability	6	Observational	83,506	47 per 100	69 per 100 (55–80)	OR 2.56 (1.40–4.70)	⊕○○○ Very low
2	Cerebral Palsy	4	Observational	3,706	5 per 100	10 per 100 (8–12)	OR 2.23 (1.72–2.96)	⊕○○○ Very low
3	Behavioural or Psychiatric Problems	4	Observational	81,439	6 per 100	15 per 100 (7–29)	OR 2.45 (1.03–5.83)	⊕○○○ Very low
**Type 1 (severe forms) compared to Type 2 (milder forms)**								
	Risk with “Type 2”	Risk with “Type 1”		
4	Cognitive Impairment or Intellectual Disability	1	Observational	5,167	46 per 100	75 per 100 (69–81)	OR 3.57 (2.62–4.86)	⊕○○○ Very low
5	Cerebral Palsy	4	Observational	1,517	10 per 100	20 per 100 (12–31)	OR 2.19 (1.20–3.80)	⊕○○○ Very low
6	Behavioural or Psychiatric Problems	2	Observational	5,500	20 per 100	41 per 100 (35–48)	OR 2.76 (2.12–3.60)	⊕○○○ Very low
**Laser compared to anti-VEGF**								
	Risk with laser	Risk with anti-VEGF		
7	Severe NDI	5	Observational	681	38 per 100	46 per 100 (34–58)	OR 1.39 (0.86–2.26)	⊕○○○ Very low
8	Cerebral Palsy	8	Observational	965	16 per 100	23 per 100 (16–31)	OR 1.55 (1.02–2.36)	⊕○○○ Very low
9	Cognitive Impairment	5	Observational	834	45 per 100	49 per 100 (35–62)	OR 1.18 (0.67–2.06)	⊕○○○ Very low

Patient—preterm infants <37 weeks. Outcomes- neurocognitive or neuropsychiatric. CI, confidence interval; OR, odds ratio. Explanations: We downgraded the evidence by three levels due to—predominant studies being observational in nature. Indirectness, inconsistency.

* The risk in the intervention group (and its 95% confidence interval) is based on the assumed risk in the comparison group and the relative effect of the intervention (and its 95% CI).

### Publication bias

Neither visual inspection of funnel plots nor the Egger test suggested publication or selection bias for the outcome of CP. The number of studies was insufficient for other outcomes to evaluate publication bias (Supplementary Material).

## Discussion

We reported the first systematic review and meta-analysis on ROP and the impact of grading and various treatment on short- and long-term neurodevelopmental and neuropsychiatric outcomes from 3 months to 18 years of age.

We found that “any ROP” in preterm infants increased the risk of cognitive impairment or intellectual disability, CP, neuropsychiatric issues, and NDI (as defined by authors) significantly compared to the “No ROP” group. Type 1 or “severe forms” of ROP (stage ≥3) increased the risk of CP and neuropsychiatric disorders significantly compared to infants with type 2 ROP or milder forms (stage <3). With regard to the modality of treatment, anti-VEGF increased the risk of CP significantly with no effect on cognitive, language, or motor impairment on unadjusted analysis. However, the significance was lost on adjusting for confounding factors, such as GA, sex sepsis, white matter injury, postnatal steroids, red blood cell transfusion, thrombocytopenia, and total parenteral nutrition. Unfortunately, these risk factors were reported in only three studies (53, 55, 60). The association of a higher risk for moderate cognitive impairment (BSID <85) after anti-VEGF treatment was present with the use of adjusted data (GA, sex, severe IVH or white matter injury, BPD, surgical NEC, sepsis, maternal education, and SNAP II score), which were reported in only two studies (58, 63).

The concern about the detrimental effects of anti-VEGF antibodies on the developing brain has been reported in previous studies (53–56, 58, 59, 60, 63). The blood concentrations of anti-VEGF can be detected for up to 2 months (64–66). Anti-VEGF acts by the destruction of astrocytes leading to reduced brain volume from animal studies (14). A previous review had found significantly lower cognitive scores in infants treated with anti-VEGF (67). The number of studies included in the meta-analysis was lower and the pooling of data from studies that used different developmental scales for assessment may have contributed to the statistical significance (51) compared to the present review. A more recent meta-analysis (68) found no difference in outcomes between the anti-VEGF treated group compared to the laser or no treatment groups. The mere association of CP with anti-VEGF therapy in the included studies where adjusted analysis was not possible could be due to the infants who received anti-VEGF being sicker, smaller, or with other comorbidities such as IVH or impaired microvascular development (BDP or IUGR). Few authors have adjusted for confounding factors such as GA and sickness level of the infants. However, major factors, such as the need for major respiratory support, NEC, and IVH, which are independent risk factors for neurodevelopmental impairment, were not consistently adjusted across all included studies. This subtle yet significant association of anti-VEGF with poor neurodevelopmental outcomes should warrant large prospective and adequately powered trials to assess its impact on the developing brain during this critical period. Therefore, we think that rigorous indication for anti-VEGF antibody treatment is mandatory until additional clinical data become available. While the indications for anti-VEGF for the treatment of ROP continue to be deliberated across the world, its popularity appears to have increased over the last two decades owing to its apparent “ease” compared to laser treatment and possibly reduced refractive error. Our data analysis raises the legitimate concern that this apparent “ease” comes with relevant side effects on the developing brain. In light of our findings, it must serve to up the ante, counsel the parents more thoroughly, and follow up with these infants more closely. The risk and benefits of the drug, and a serious consideration to rule out alternative laser therapy, must be declared to the parents until more evidence or stronger associations are found to prove the contrary.

The comparison of “anti-VEGF plus laser” vs. “anti-VEGF” was limited. Our data imply that additional laser treatment did not increase the risk for adverse neurodevelopmental outcomes by anti-VEGF treatment *per se*. This fact will be important in assessing any risk and benefit when the persistent peripheral avascular retina, recurrence of ROP, and incomplete regression are encountered, and may suggest that laser rather than a second anti-VEGF injection may be systemically safer.

The association between ROP and neurodevelopmental outcomes has been reported for the past two decades in previous studies (36, 38, 41). ROP has been associated with reduced head circumference, cerebellar volume, and unmyelinated white matter volume in previous studies (70, 71). The CAP trial has shown that severe ROP increases the risk of poor cognitive and motor outcomes by three- to fourfold (10). A large database study involving 79,373 infants showed that infants needing treatment for ROP are at increased risk for intellectual disabilities, psychiatric and behavioural disorders, speech and language impairment, and ASDs (12, 13, 37). However, several other studies have found no association between ROP and adverse neurodevelopmental outcomes, and any deviations of development are attributed to prematurity as such but not with ROP (26, 28, 32, 40). Although contrasting evidence, the consistent association of ROP and poor neurodevelopmental outcomes could not be merely incidental and its role in causation needs to be further explored. The plausible explanation for the causal role is attributed to the elevated inflammatory markers (46, 72–75), deficiency of insulin-like growth factor (IGF-1) (76–78), hyperoxia or fluctuating oxygen levels (79, 80) in both ROP and brain injury, and combinations thereof.

### Strengths and limitations

The strength of this meta-analysis was that we used a broad comprehensive search strategy to include articles with all possible neurodevelopmental outcomes from infancy until adulthood. The included studies in the present review measured different outcomes at different time points using various developmental scales. Most studies used the definition of “severe ROP” as per the ETROP trial; however, some studies used more pragmatic definitions, such as stage ≥3 or those needing treatment. Few studies reported outcomes using ICD codes retrospectively. This heterogeneity is inherent to the development of clinical care over a period of time.

The majority of the studies were observational and heterogenous, and the non-uniformity of definitions used to define developmental disorder(s) and various developmental scales to measure the outcome adds to the limitations of the study. The analysis of different treatments on neurodevelopmental outcomes using adjusted data was also a strength in helping to define subsequent clinical questions on the indication of anti-VEGF treatment.

## Conclusion

Our data support the hypothesis that ROP in preterm infants may be an independent indicator for impaired microvascular development in the brain resulting in poor neurodevelopment outcomes. Clinical data analyses and trials need to address the question of the long-term safety of anti-VEGF treatment.

## Data Availability

The original contributions presented in the study are included in the article/**Supplementary Material**, further inquiries can be directed to the corresponding author.
