# Supplementary material for: Retinopathy of prematurity and neurodevelopmental outcomes in preterm infants: A systematic review and meta-analysis
Source: Front Pediatr. 2023 Mar 15;11:1055813. doi: 10.3389/fped.2023.1055813 (PMC10050340; doi:10.3389/fped.2023.1055813)

Online Supplemental File

Table of Contents

Supp file 1: Search Strategy - ROP and Adverse neurodevelopmental Outcomes **2**

Supp file 2: List of important excluded studies **3**

Supp file 3 : Forest plots: 'Any ROP' versus 'No ROP' **5**

Supp file 4: Forest plots: 'Type 1' versus 'Type 2' **8**

Supp file 5: Forest plots: Laser versus 'Anti-VEGF' **11**

Supp file 6: ROB 2.0 tool for RCT studies for all included studies **17**

Supp file 7: New-Castle Ottawa Scale score for all included studies **19**

Supp file 8: Funnel Plot for Publication bias **24**

Supp file 9: Meta-regression scatter plots **25**

Supp file 1: Search Strategy - ROP and Adverse neurodevelopmental Outcomes

Search performed on : 8.4.22

PubMed:

P: #1 Neonate OR Neonatal* OR Newborn OR Preterm OR Premature* OR Premature OR Infant OR ‘Low birth weight’ OR ‘very low birth weight’

AND

O: #2 ‘Retinopathy of prematurity’ OR ‘ROP’ OR retinopathy* OR ‘retrolental fibroplasia’

AND

O: **#3** ‘Neurodevelopmental outcome’ OR ‘Neurodevelopment* outcome’ OR ‘Neuropsychiatric outcome’ OR ‘Neuropsychiatry* outcome’ OR ‘Cognitive’ OR ‘development*’

Search: #1 AND #2 AND #3

PubMed: English: 407, All Languages: 416

Supp file 2: List of important excluded studies

Study ID	Reason for Exclusion
Opsina et al	No data for controls (No ROP data)
Schimdt 2003	ROP outcome data not available
Stahl 2022 (CARE-ROP)	No data for controls (No ROP data)
Naravane 2022	Case series
Jacobson 1998	No data for controls (No ROP data)
Holsti 2018	ROP outcome data not available
Cevik 2020	No data for controls (No ROP data)
Castellanos 2013	No data for controls (No ROP data)

Bowen 1993	Outcomes for all ELBW, no ROP data specifically available
Yazici 2022	BPD study, no ROP outcome data available

Supp file 3 : Forest plots: ‘Any ROP’ versus ‘No ROP’

1: Cognitive Composite score BSID III (Combined time points 18-48 months)
2a : Language Composite score – BSID III (18-24 months) ; 2b Language Composite score – BSID III (combined time points 18-48 months)
3a.Motor Composite score – BSID III (18-24 months); 3bMotor Composite score – BSID III (combined time points 18-48 months)
4: Neurodevelopmental impairment (defined by authors)

1

Study name	Time point	Statistics for each study			
		Std diff in means	Lower limit	Upper limit	p-Value
Ahn 2021	18-24 months	-0.398	-0.838	0.042	0.076
Altendahl 2021	18-24 months	-0.485	-0.825	-0.144	0.005
Altendahl 2021	25-36 months	-0.555	-1.075	-0.034	0.037
Brumbaugh 2021	18-24 months	-3.462	-3.743	-3.180	0.000
Drost 2018	18-24 months	-0.112	-0.733	0.508	0.722
Fan 2021	18-24 months	0.379	0.053	0.705	0.023
		-0.777	-2.167	0.613	0.273

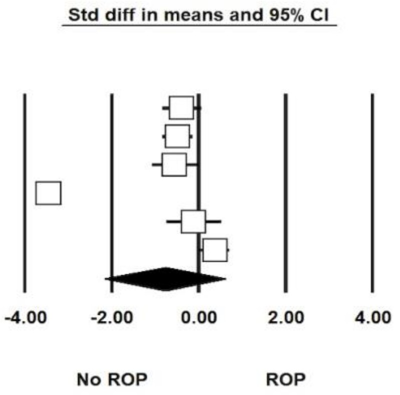

2a

Study name	Std diff in means	Lower limit	Upper limit	p-Value
Brumbaugh 2021	-0.749	-0.939	-0.560	0.000
Ahn 2021	-0.257	-0.695	0.181	0.250
Altendahl 2021	-0.414	-0.753	-0.075	0.017
Fan 2021	-0.232	-0.557	0.092	0.160
	-0.443	-0.730	-0.157	0.002

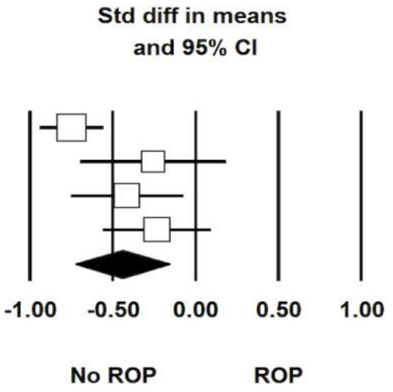

2b

Study name	Time point	Std diff in means	Lower limit	Upper limit	p-Value
Ahn 2021	18-24 months	-0.257	-0.695	0.181	0.250
Altendahl 2021	18-24 months	-0.414	-0.753	-0.075	0.017
Altendahl 2021	25-36 months	-0.576	-1.112	-0.040	0.035
Brumbaugh 2021	18-24 months	-0.749	-0.939	-0.560	0.000
Fan 2021	18-24 months	-0.232	-0.557	0.092	0.160
		-0.466	-0.710	-0.223	0.000

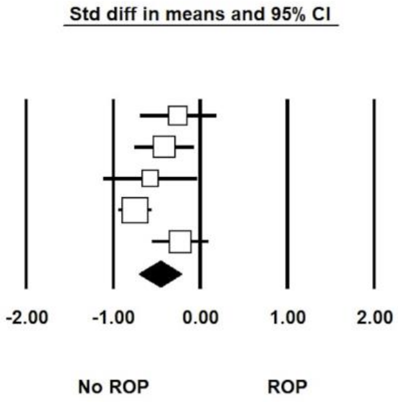

3a

Study name	Std diff in means	Lower limit	Upper limit	p-Value
Brumbaugh 2021	-0.149	-0.333	0.034	0.111
Ahn 2021	-0.350	-0.789	0.089	0.119
Altendahl 2021	-0.537	-0.878	-0.195	0.002
Fan 2021	-0.318	-0.643	0.007	0.055
	-0.292	-0.469	-0.116	0.001

Statistics for each study

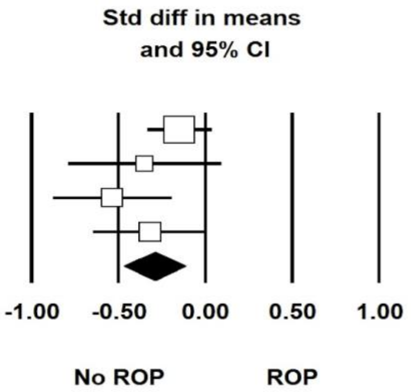

3b

Study name	Time point	Std diff in means	Lower limit	Upper limit	p-Value
Ahn 2021	18-24 months	-0.350	-0.789	0.089	0.119
Altendahl 2021	18-24 months	-0.537	-0.878	-0.195	0.002
Altendahl 2021	25-36 months	-0.386	-0.902	0.129	0.142
Brumbaugh 2021	18-24 months	-0.149	-0.333	0.034	0.111
Fan 2021	18-24 months	-0.318	-0.643	0.007	0.055
		-0.283	-0.428	-0.138	0.000

Statistics for each study

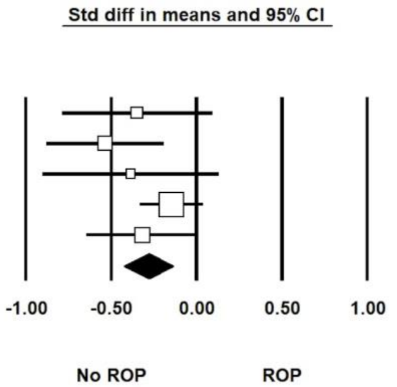

4

Study name	Odds ratio	Lower limit	Upper limit	p-Value
Borregas 2018	3.841	2.025	7.285	0.000
Hungerford	16.943	4.444	64.589	0.000
Ricci	2.114	0.699	6.395	0.185
Beligere 2015	0.081	0.005	1.428	0.086
Msall 2000	6.193	3.245	11.817	0.000
	3.836	1.612	9.127	0.002

Statistics for each study

Odds ratio and 95% CI

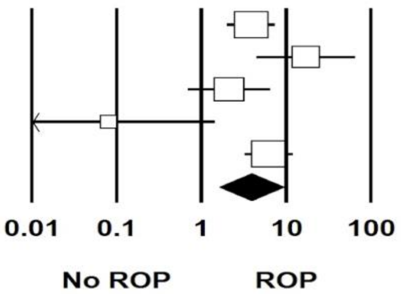

Supp file 4: Forest plots: ‘Type 1’ versus ‘Type 2’

1: Cognitive Composite score BSID III (combined time points 18-48 months)
2a : Language Composite score – BSID III (18-24 months) ; 2b Language Composite score – BSID III (combined time points 18-48 months)
3a.Motor Composite score – BSID III (18-24 months); 3b Motor Composite score – BSID III (combined time points 18-48 months)

1

Study name	Time point	Std diff in means	Lower limit	Upper limit	p-Value
		Statistics for each study			
Altendahl 2021	18-24 months	-0.375	-0.925	0.175	0.181
Altendahl 2021	25-36 months	-0.416	-1.199	0.366	0.297
Brumbaugh 2021	18-24 months	-3.625	-4.005	-3.245	0.000
Fan 2021	18-24 months	-0.275	-0.751	0.202	0.258
Glass 2017	18-24 months	-0.916	-1.479	-0.353	0.001
Ahn 2020	18-24 months	-0.439	-0.991	0.112	0.118
Chang 2019	18-24 months	0.340	-0.272	0.952	0.276
		-0.824	-2.035	0.387	0.182

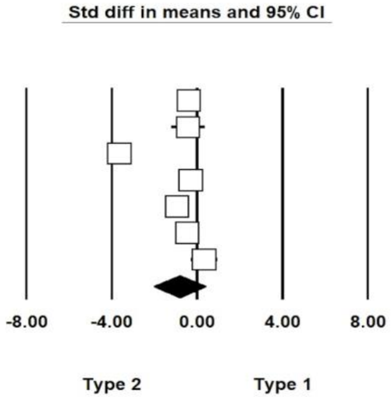

2a

Study name	Std diff in means	Lower limit	Upper limit	p-Value
	Statistics for each study			
Ahn 2020	-0.067	-0.614	0.480	0.810
Altendahl 2021	-0.291	-0.861	0.279	0.317
Brumbaugh 2021	-3.133	-3.487	-2.779	0.000
Fan 2021	-0.060	-0.534	0.415	0.805
Glass 2017	-0.289	-0.836	0.259	0.301
Chang 2019	0.002	-0.607	0.611	0.995
	-0.647	-1.901	0.606	0.311

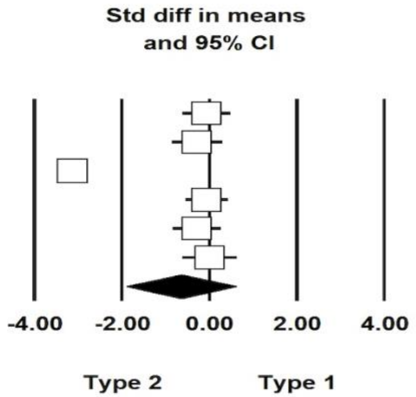

2b

Study name	Time point	Std diff in means	Lower limit	Upper limit	p-Value
		Statistics for each study			
Ahn 2020	18-24 months	-0.067	-0.614	0.480	0.810
Altendahl 2021	18-24 months	-0.291	-0.861	0.279	0.317
Altendahl 2021	25-36 months	-0.121	-0.903	0.660	0.761
Brumbaugh 2021	18-24 months	-3.133	-3.487	-2.779	0.000
Fan 2021	18-24 months	-0.060	-0.534	0.415	0.805
Glass 2017	18-24 months	-0.289	-0.836	0.259	0.301
Chang 2019	18-24 months	0.002	-0.607	0.611	0.995
		-0.575	-1.703	0.553	0.318

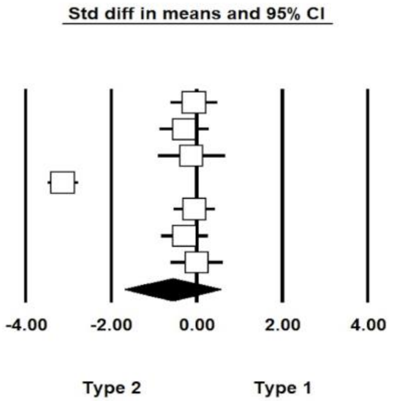

3a

Study name	Std diff in means	Lower limit	Upper limit	p-Value
	Statistics for each study			
Ahn 2020	-0.255	-0.804	0.293	0.361
Altendahl 2021	-0.159	-0.727	0.409	0.584
Chang 2019	-0.307	-0.919	0.304	0.324
Brumbaugh 2021	-4.117	-4.525	-3.709	0.000
Fan 2021	-0.255	-0.732	0.221	0.293
Glass 2017	-0.772	-1.330	-0.214	0.007
	-0.983	-2.464	0.497	0.193

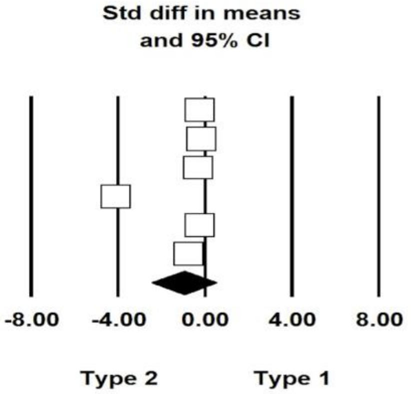

3b

Study name	Time point	Std diff in means	Lower limit	Upper limit	p-Value
		Statistics for each study			
Ahn 2020	18-24 months	-0.255	-0.804	0.293	0.361
Altendahl 2021	18-24 months	-0.159	-0.727	0.409	0.584
Altendahl 2021	25-36 months	-0.474	-1.258	0.311	0.236
Chang 2019	18-24 months	-0.307	-0.919	0.304	0.324
Brumbaugh 2021	18-24 months	-4.117	-4.525	-3.709	0.000
Fan 2021	18-24 months	-0.255	-0.732	0.221	0.293
Glass 2017	18-24 months	-0.772	-1.330	-0.214	0.007
		-0.913	-2.234	0.409	0.176

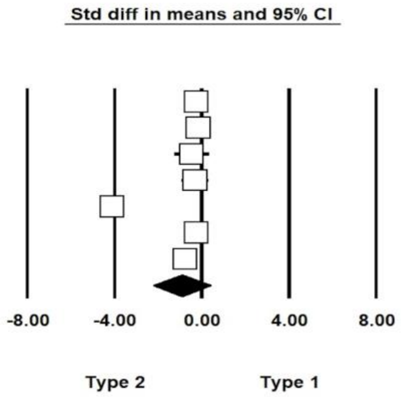

Supp file 5: Forest plots: Laser versus 'Anti-VEGF'

1: Cognitive Composite score BSID III (combined time points 18-48 months AND scales)
2a : Language Composite score – BSID III (18-24 months) ; 2b Language Composite score – BSID III (combined time points 18-48 months)
3a. Motor Composite score – BSID III (18-24 months); 3b. Motor Composite score – BSID III (combined time points 18-48 months)
4. Moderate -Severe NDI 4b. Severe NDI
5. Cognitive impairment
6a. Moderate language impairment- BSID III; 6b. Severe language impairment- BSID III;
7a. Moderate motor impairment- BSID III; 7b. Severe motor impairment- BSID III;

1

Study name	Std diff in means	Lower limit	Upper limit	p-Value
	Statistics for each study			
Kennedy 2018	0.433	-0.566	1.432	0.396
Morin 2016	-0.173	-0.599	0.254	0.427
Natarajan 2019	-0.144	-0.354	0.066	0.178
Raghuram 2019	-0.247	-0.766	0.273	0.352
Rodriguez 2019	0.015	-0.835	0.865	0.972
Zayek 2020	0.245	-0.244	0.734	0.325
Lien 2016	-0.882	-1.567	-0.196	0.012
Celik 2021	-0.335	-1.002	0.332	0.325
Arima 2020	-0.436	-1.053	0.180	0.165
	-0.172	-0.353	0.010	0.064

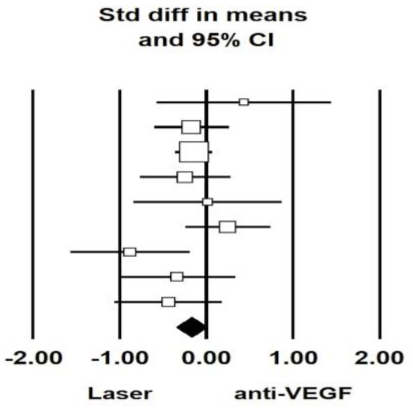

2a

Study name	Std diff in means	Lower limit	Upper limit	p-Value
	Statistics for each study			
Kennedy 2018	0.218	-0.773	1.208	0.667
Morin 2016	-0.155	-0.581	0.272	0.477
Natarajan 2019	-0.029	-0.240	0.182	0.790
Raghuram 2019	-0.131	-0.681	0.420	0.642
Rodriguez 2019	-0.047	-0.898	0.803	0.913
Zayek 2020	0.099	-0.388	0.587	0.690
Arima 2020	-0.773	-1.401	-0.145	0.016
	-0.082	-0.239	0.075	0.307

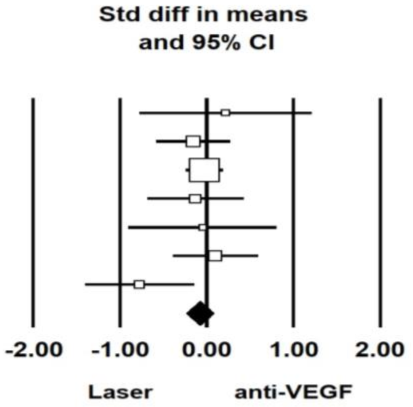

2b

Study name	Time point	Std diff in means	Lower limit	Upper limit	p-Value
Kennedy 2018	18-24 months	0.218	-0.773	1.208	0.667
Morin 2016	18-24 months	-0.155	-0.581	0.272	0.477
Natarajan 2019	18-24 months	-0.029	-0.240	0.182	0.790
Raghuram 2019	18-24 months	-0.131	-0.681	0.420	0.642
Rodriguez 2019	18-24 months	-0.047	-0.898	0.803	0.913
Zayek 2020	18-24 months	0.099	-0.388	0.587	0.690
Celik 2021	18-24 months	0.096	-0.567	0.760	0.776
Celik 2021	25-36 months	0.096	-0.567	0.760	0.776
		-0.022	-0.175	0.132	0.783

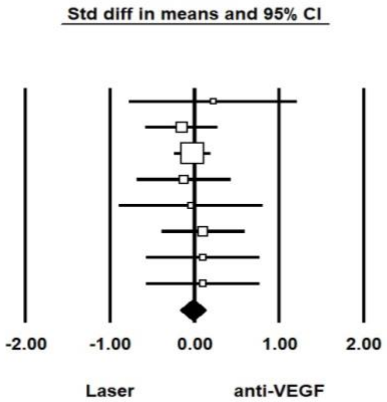

3a

Study name	Std diff in means	Lower limit	Upper limit	p-Value
Kennedy 2018	0.028	-0.960	1.016	0.956
Lien 2016	-0.774	-1.454	-0.094	0.026
Morin 2016	-0.586	-1.018	-0.154	0.008
Natarajan 2019	-0.074	-0.285	0.137	0.491
Raghuram 2019	-0.288	-0.852	0.276	0.317
Rodriguez 2019	0.169	-0.682	1.021	0.697
Zayek 2020	0.306	-0.184	0.796	0.221
	-0.185	-0.460	0.089	0.186

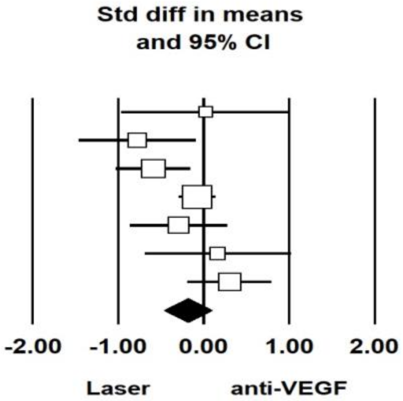

3b

Study name	Time point	Std diff in means	Lower limit	Upper limit	p-Value
		Statistics for each study			
Kennedy 2018	18-24 months	0.028	-0.960	1.016	0.956
Lien 2016	18-24 months	-0.774	-1.454	-0.094	0.026
Morin 2016	18-24 months	-0.586	-1.018	-0.154	0.008
Natarajan 2019	18-24 months	-0.074	-0.285	0.137	0.491
Raghuram 2019	18-24 months	-0.288	-0.852	0.276	0.317
Rodriguez 2019	18-24 months	0.169	-0.682	1.021	0.697
Zayek 2020	18-24 months	0.306	-0.184	0.796	0.221
Celik 2021	18-36 months	-0.004	-0.668	0.659	0.990
		-0.166	-0.410	0.078	0.182

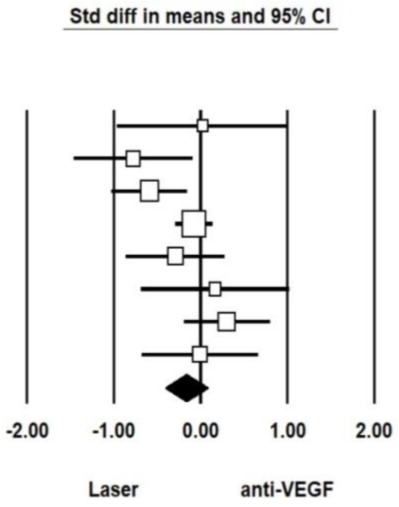

4

Study name	Odds ratio	Lower limit	Upper limit	p-Value
	Statistics for each study			
Celik 2021	0.684	0.180	2.596	0.577
Chen 2017	0.870	0.080	9.461	0.909
Morin 2016	2.736	1.016	7.373	0.047
Raghuram 2019	1.625	0.542	4.869	0.386
Zayek 2020	0.794	0.261	2.415	0.685
	1.341	0.775	2.320	0.294

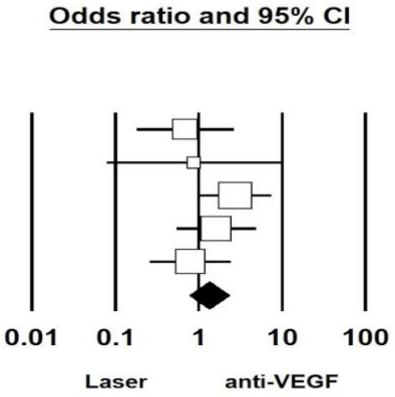

5

Study name	Odds ratio	Lower limit	Upper limit	p-Value
Zayek 2020	0.590	0.217	1.610	0.303
Natarajan 2019	1.142	0.750	1.740	0.536
Celik 2021	1.571	0.399	6.182	0.518
Raghuram 2019	2.188	0.801	5.976	0.127
Morin 2016	2.692	1.125	6.445	0.026
	1.392	0.857	2.260	0.181

(Columns Odds ratio to p-Value: Statistics for each study)

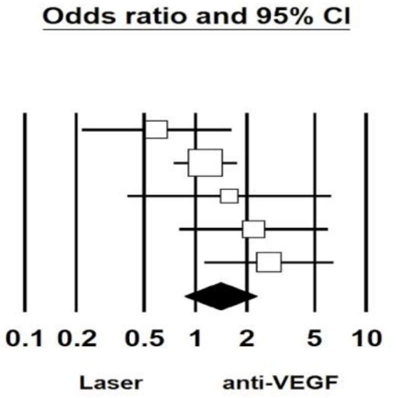

6a

Study name	Odds ratio	Lower limit	Upper limit	Z-Value	p-Value
Celik 2021	1.364	0.350	5.312	0.447	0.655
Hye 2022	0.495	0.254	0.964	-2.066	0.039
Morin 2016	2.233	0.894	5.576	1.721	0.085
Natarajan 2019	1.561	1.023	2.383	2.064	0.039
Zayek 2020	1.088	0.409	2.892	0.168	0.867
	1.176	0.672	2.057	0.567	0.570

(Columns Odds ratio to p-Value: Statistics for each study)

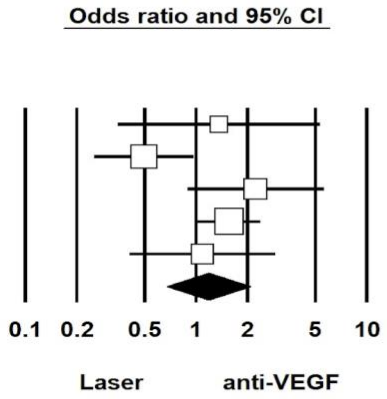

6b

Study name	Odds ratio	Lower limit	Upper limit	p-Value
Hye 2022	0.807	0.350	1.859	0.614
Morin 2016	1.786	0.746	4.277	0.193
	1.187	0.545	2.586	0.666

Statistics for each study — Odds ratio and 95% CI

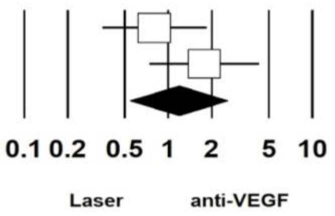

7a

Study name	Odds ratio	Lower limit	Upper limit	p-Value
Celik 2021	1.278	0.307	5.320	0.736
Natarajan 2019	1.046	0.669	1.635	0.845
	1.065	0.695	1.631	0.774

Statistics for each study — Odds ratio and 95% CI

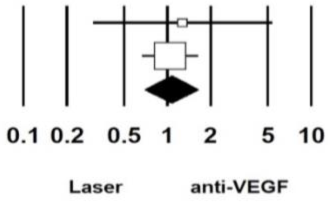

7b

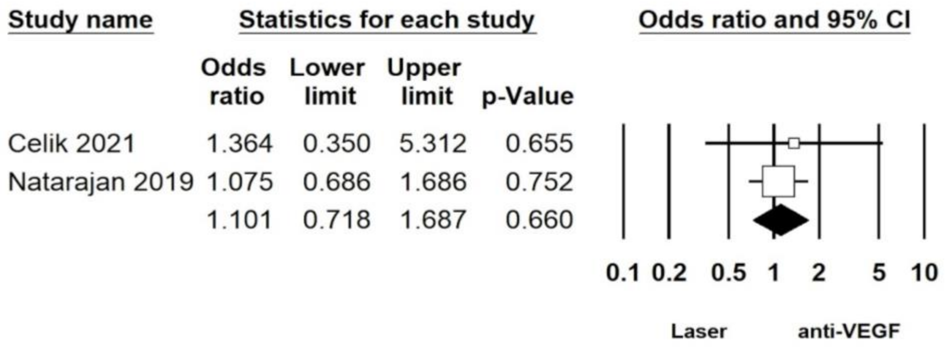

Supp file 6: ROB 2.0 tool for RCT studies for all included studies

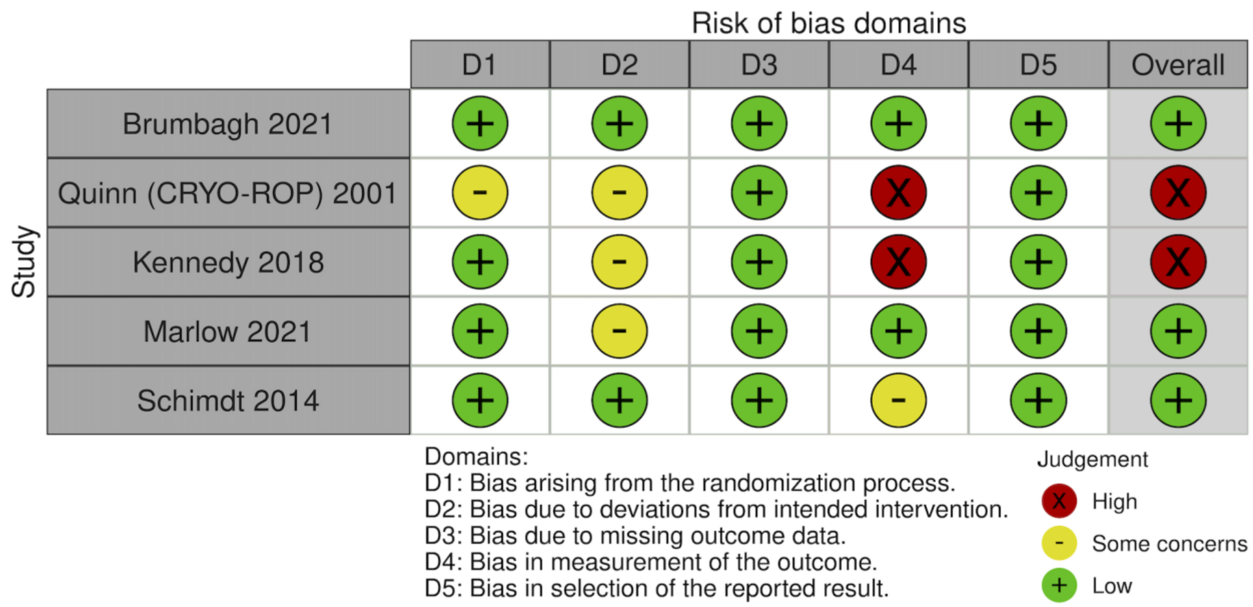
Risk of bias domains
D1
D2
D3
D4
D5
Overall
Study
Brumbagh 2021
Quinn (CRYO-ROP) 2001
Kennedy 2018
Marlow 2021
Schimdt 2014
Domains:
D1: Bias arising from the randomization process.
D2: Bias due to deviations from intended intervention.
D3: Bias due to missing outcome data.
D4: Bias in measurement of the outcome.
D5: Bias in selection of the reported result.
Judgement
High
Some concerns
Low

Supp file 7: New-Castle Ottawa Scale score for all included studies

	**Study ID**	**Selection (max 4)**				**Outcome (max 3)**			**Comparative (max 2)**		**Total Score (max 9)**	**Interpretation**
		Representativeness of the exposed cohort (1)	Ascertainment of exposure (1)	Demonstration that outcome of interest was not present at start of study (1)	Selection of non-exposed amongst cohort (i.e., sample drawn from same neonatal population/ nicu) (1)	Assessment of outcome (1)	Was follow-up long enough for outcomes to occur (1)	Adequacy of follow up of cohorts (1)	Study controls for GA and Sex (1)	Study controls for additional factors (1)		> /=6 (Good quality) 4,5 (fair quality) 1,2,3 (poor quality)
1	Ahn 2021	1	1	1	0	1	1	1	0	0	6	good
2	Altendahl etal 2021	1	1	1	1	1	0	1	0	1	7	good
3	Arima et al 2020	1	1	1	1	1	0	1	0	1	6	good

4	Allred et al 2014	1	1	1	1	1	1	1	1	1	9	good
5	Borregas etal 2018	1	1	1	0	0	0	0	1	1	5	fair
6	Bae etal 2021	1	1	1	1	1	0	1	1	0	7	good
7	Bohm etal 2002	1	1	1	0	1	0	1	0	0	5	fair
8	Chen et al 2017	1	1	1	0	1	1	1	1	0	7	good
9	Chen et al 2003	1	1	1	1	1	1	1	0	0	7	good
10	Celik et al 2021	1	1	1	1	1	1	1	1	0	8	good
11	Jin choi etal 2022	1	1	1	1	1	1	0	0	0	6	good
12	Chang et al 2019	1	1	1	1	1	0	0	1	1	7	good
13	Chou et al 2020	1	1	1	1	1	1	1	0	1	8	good
14	Drost et al 2018	1	1	1	1	0	1	1	0	0	6	good
15	Fan 2021	1	1	1	1	1	1	0	1	0	7	good
16	Glass et al 2017	1	1	1	1	1	0	1	1	0	7	good

17	Hungerford etal 1986	1	1	1	0	1	0	0	1	0	5	fair
18	Hye et al 2022	1	1	1	1	1	1	1	1	0	8	good
19	Holsti 2018	1	1	1	0	1	1	1	0	0	6	Good
20	Jacobson et al 2020	1	1	1	1	0	1	1	1	1	8	good
21	Kennedy 2018	1	1	0	1	1	1	1	1	0	7	good
22	Lien 2016	1	1	1	1	1	1	1	1	0	8	good
23	Morin etal 2016	1	1	1	0	1	0	1	0	1	6	good
24	Moujahed et al 2020	1	1	1	1	1	0	0	1	1	7	good
25	Msall et al 2000	1	1	1	1	1	1	0	0	1	7	good
26	Natarajan etal 2019	1	1	1	0	1	1	1	1	0	7	good
27	Raghuram et al 2019	1	1	1	1	1	1	0	0	1	7	good
28	Ricci etal2020	1	0	1	1	1	0	1	1	0	6	good

29	Sugimoto et al 1998	1	1	1	0	0	0	1	1	0	5	fair
30	Stephenson etal 2007	1	1	1	1	1	1	1	0	0	7	good
31	Todd et al 2012	1	1	0	0	1	0	1	1	1	6	good
32	Zayek et al 2020	1	1	1	1	1	0	1	0	1	7	good
33	Zhang et al 2020	1	1	1	1	0	1	1	0	0	6	good

Supp file 8: Funnel Plot for Publication bias

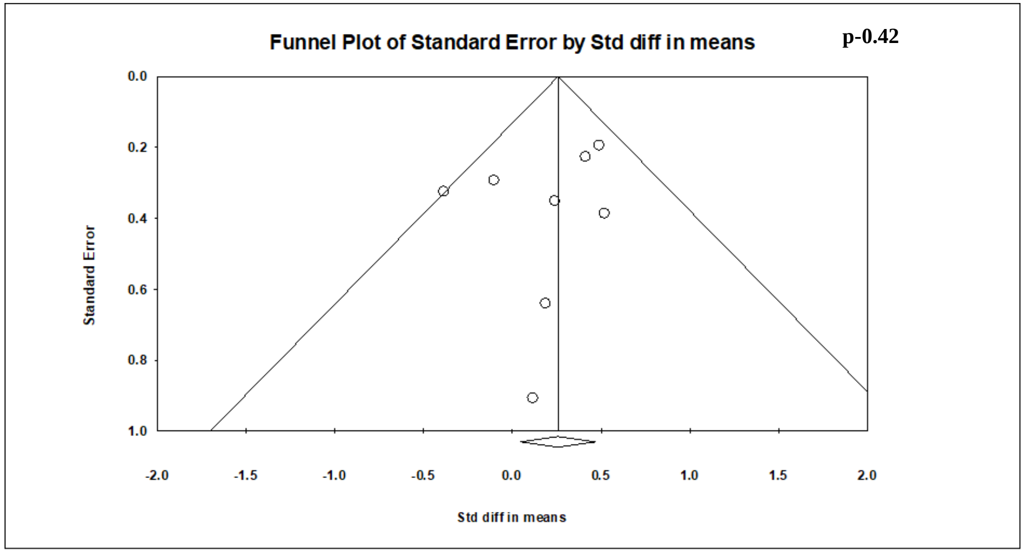

Supp file 9: Meta-regression scatter plots

a. Cerebral Palsy (anti-VEGF versus laser)

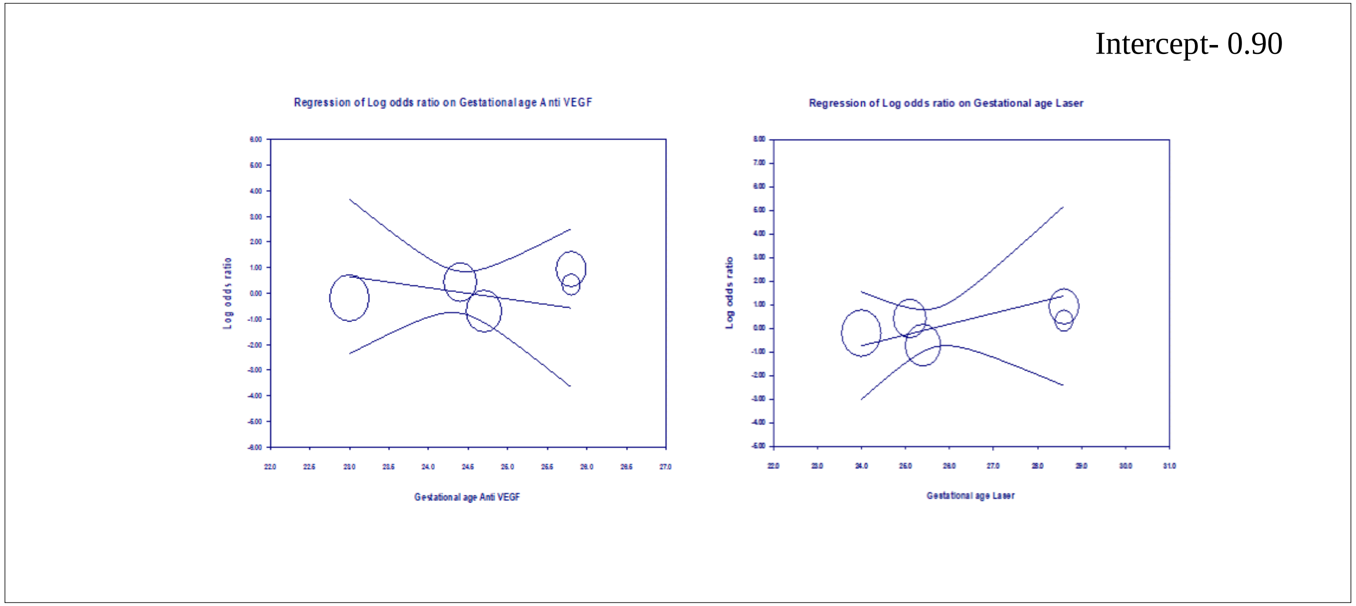

b. Cognitive composite score (anti-VEGF versus laser)

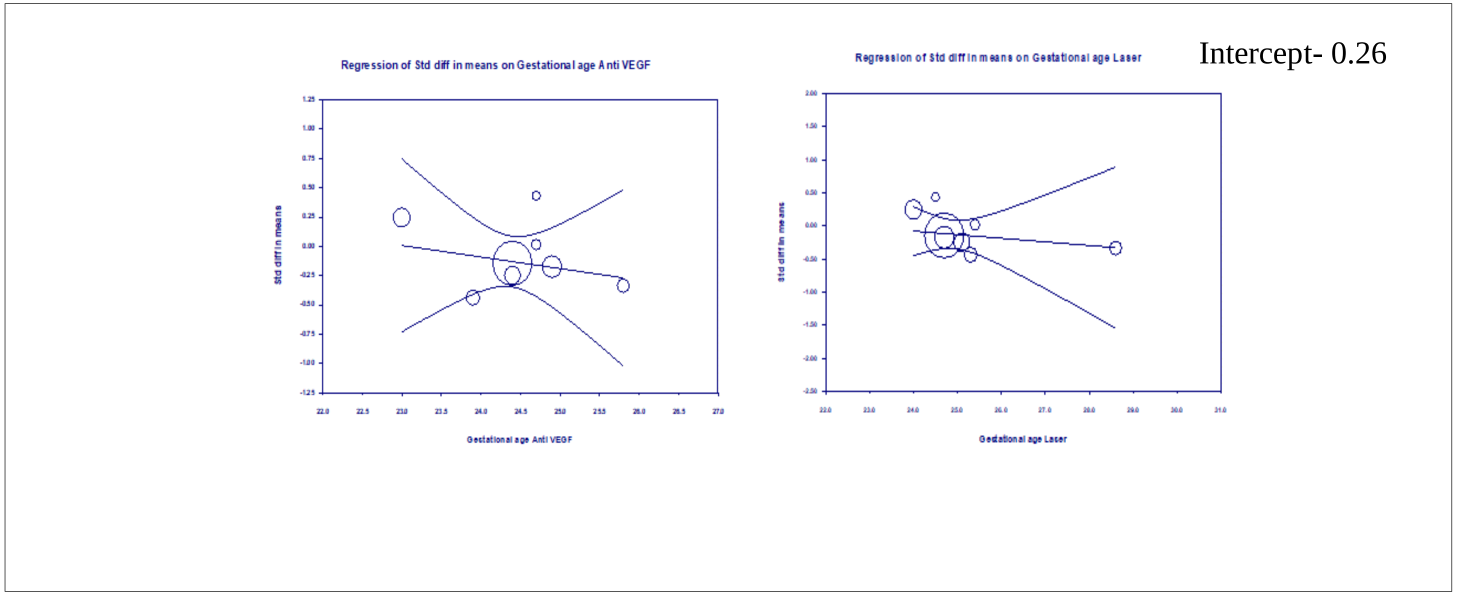

c. Language composite score (anti-VEGF versus laser)

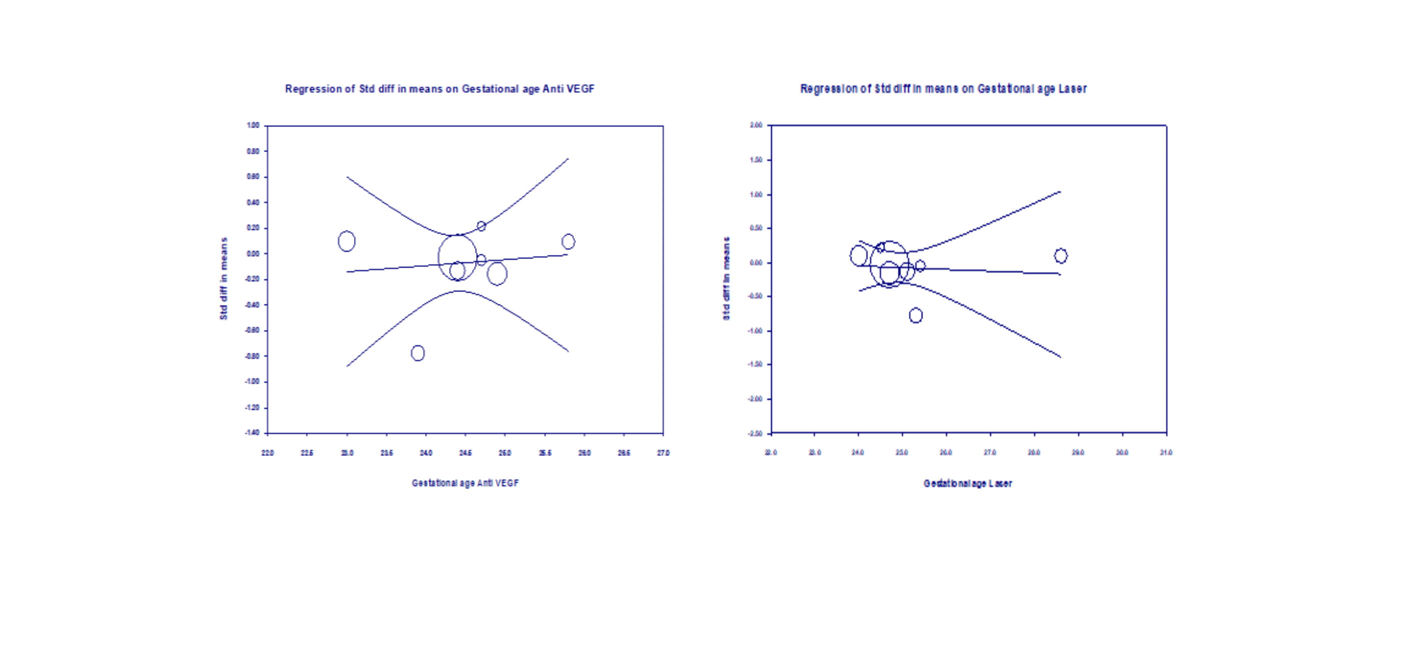

d. Motor composite score (anti-VEGF versus laser)

Intercept- 0.08

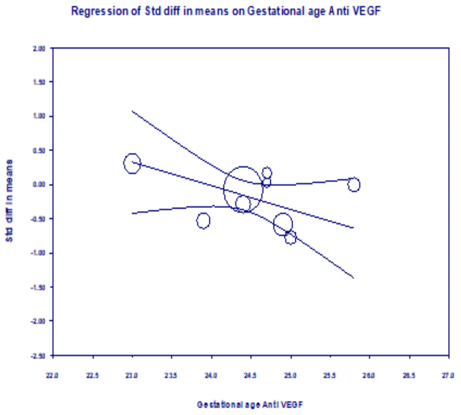

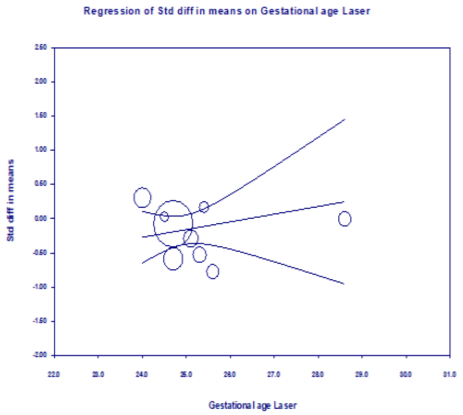

Supplement: Supplementary file 1 [file Datasheet1.pdf]
